# Long-Term Impact of Historical BCG Vaccination Policies on Parkinson’s Disease Risk: A Hypothesis-Generating Retrospective Cohort Analysis

**DOI:** 10.3390/biomedicines14091945

**Published:** 2026-08-29

**Authors:** Benjamin Y. Klein, Ofer N. Gofrit, Charles L. Greenblatt

**Affiliations:** 1 Department of Microbiology and Molecular Genetics, Hebrew University of Jerusalem, Jerusalem 9112102, Israel; charlesg@ekmd.huji.ac.il; 2 Department of Urology, Hadassah Medical Center, Ein-Kerem, Jerusalem 9112001, Israel; ogofrit@gmail.com

**Keywords:** BCG, sex differences, innate immunity, neurodegeneration, aging, birth cohorts

## Abstract

**Objectives**: Adults exposed to the Bacillus Calmette–Guérin (BCG) vaccine demonstrate a reduced incidence of Parkinson’s disease (PD). This retrospective study evaluated whether early-life BCG vaccination predicts a long-term reduction in PD incidence during aging. **Methods**: Published age-adjusted incidence rates (AAIRs) of PD (2002–2021) were structured into single-age birth cohorts. Missing values were calculated via interpolation and shuffling simulations. Cohorts were categorized into three historical BCG policy periods: unvaccinated controls (born 1945–1949), vaccinated at age 12 (born 1950–1954), and vaccinated as newborns (born 1955–1959). AAIRs were compared at identical cross-sectional ages (57 and 62 years), applying an environmental drift penalty to control for temporal improvements. **Results**: In men, the age 12 policy showed a 21.8% AAIR reduction at age 57, which attenuated to 4.4% by age 62; the newborn policy showed a sustained reduction (25.8% at age 57; 20.4% at age 62). In women, the age 12 policy yielded a 67.5% reduction at age 57 (33.1% at age 62), while the newborn policy showed a 56.8% reduction at age 57 (44.0% at age 62). **Conclusions**: Early-life BCG vaccination is associated with reduced later-life PD incidence, displaying a profound sex-dependent effect favoring females. Newborn vaccination provides long-term resistance as well.

## 1. Introduction

Retrospective analysis demonstrated that intravesical exposure to the Bacillus Calmette–Guérin (BCG) vaccine in adults with non-muscle invasive bladder cancer (NMIBC) protects against the development of sporadic Alzheimer’s disease (AD) [1,2]. Similar exposure to BCG also protected against Parkinson’s disease (PD) [2]. Both are neurodegenerative diseases of old age with distinct characteristics; both develop covert pathophysiological signs long before overt signs appear, leading to clinical diagnosis [3]. PD may be diagnosed 5–10 years earlier than AD (at age 40 or 50–64) [4,5], whereas sporadic AD is diagnosed at age 65–70 at the earliest, as in the BCG retrospective study [1]. The prophylactic effect against AD and PD observed with intravesical BCG instillation in NMIBC patients implies that at least part of the common pathology responds to BCG vaccine therapy. One of the hallmarks of AD is the aggregation of the β-amyloid protein and its fragments, forming plaques, and the highly phosphorylated tau protein, forming tangles [6]. This suggests that part of the pathology of AD might involve a disrupted unfolded protein response (UPR) to endoplasmic reticulum (ER) stress caused by misfolded newly synthesized proteins [7,8,9]. By comparing peripheral blood mononuclear cells (PBMCs) isolated from NMIBC and from melanoma patients before and after BCG therapy, BCG enhances the UPR cascade in half of the tested individuals [10], including melanoma patients treated with BCG as an adjuvant [11]. Exploring the impact of BCG on UPR in peripheral immunocytes is justified by their involvement in AD pathology and their protective role against AD development [12,13,14,15]. The disrupted UPR cascade also appears among the hallmarks of PD pathology, reflected in α-synuclein [16,17] and in gut tissue, and is involved in amygdala dysfunction in AD [3]. BCG is involved in additional protective mechanisms (see Section 4). Symptoms of PD can appear late in AD patients, and vice versa, symptoms of AD can appear late in the course of PD; these inverted phenomena have been hypothesized as if PD and AD are two faces of one neurodegenerative disease of the elderly [3]. The BCG vaccine was developed at the beginning of the twentieth century to immunize against tuberculosis [18], its off-target effect on decreasing the rate of AD and PD [1,2,19] and improving cognitive function [20] was described in the context of adult treatment of cancer with the vaccine; a separate question is whether early-life vaccination may have an impact on the development of diseases associated with aging, much later in life. This could be the case if newborn vaccination is detectable later by a delayed-type hypersensitivity skin reaction to the Mantoux test, upon aging. Evidence for such a response 60 years after newborn vaccination has been documented [21]. In countries where mandatory newborn BCG vaccination was implemented in the 1950s, PD incidence recorded during the second decade of the 21st century is suitable for analyzing BCG’s impact on PD incidence. To analyze the impact on AD incidence, data from the far future may be suitable. However, obtaining a database of disease incidence is challenging [22], and mere accessibility to these data is crucial. This leaves published population studies of diseases as the last option for the garden-variety molecular biologist. Here, an attempt is made to construct the missing PD incidence data in gaps between mean incidence values published in PD population studies. The results of reconstituting blank incidence values demonstrate that, beyond the decline in PD incidence, due to unknown factors, early-life vaccination per se may reduce PD incidence. Decline in PD incidence along age cohorts in Germany may show an additional decline in BCG-vaccinated individuals early in life.

Fink et al. [23] analyzed the age-specific incidence rates of PD among members of a German health organization (AOK) during two periods, 2006–2007 and 2008, against 2016, 2017, and 2018. They showed a decline in age-specific incidence comparing the late to the early period. Identical ages along the trajectories of birth cohorts before newborn vaccination was practiced were compared with the same ages on trajectories from 1952 and 1953. In 1951/52, BCG vaccination programs were extended. In the Eastern German Democratic Republic (GDR), mandatory newborn BCG vaccination was introduced in 1953, reaching 95% coverage by the time of the two countries’ reunification. In contrast, in West Germany, the vaccination was voluntary, resulting in only 25% coverage [24]. A recent study in Israel [25] showed a decline in PD incidence with an extended date range compared to the German study, which we use here to analyze a possible effect on future PD incidence.

## 2. Materials and Methods

### 2.1. General

Treatment of adult populations with the BCG vaccine to estimate its impact on the development of neurodegenerative diseases, such as Alzheimer’s or Parkinson’s disease, can be conducted in adults as a prospective study. Prospective studies are likely triggered by several retrospective studies, which have shown that exposure to the BCG vaccine reduces the incidence of AD [1,2] and PD [2]. A crucial question is whether newborn or early-age BCG vaccination is sufficiently sustainable to prevent such diseases as one ages. This kind of study can by no means be conducted prospectively, as the investigators’ grandchildren will have to summarize the results, as rarely exemplified [21]. The remaining solution is to conduct retrospective studies under restrictive conditions, for example, in a non-immigration country where past mandatory BCG vaccination programs are well documented. Under this condition, one can use the annual number of new cases over a prolonged period, such as at least 10 consecutive years, and use live births as the denominator, suitable for the year of birth of diagnosed populations. Obtaining a consecutive year-by-year database of newly diagnosed individuals is next to impossible for many investigators; therefore, one must resort to alternatives as follows.

### 2.2. Repurposing Published Data

A study conducted in Israel [25] showed a decline in Parkinson’s disease over 20 diagnostic years from 2002 to 2021. In their study, as shown in Table 1 [25], they reported age-adjusted incidence rates (AAIRs) for each of the 20 diagnostic years, a 57/100,000 age-matched population (in 2002), with a low rate of 20.23/100,000 late in 2021. Each of these changes in incidence relates to the mean incidence given for each age quintet in their study, as shown in Table 2 [25]. To construct a detailed matrix in which each age-year group is assigned to an AAIR, it is necessary to assign AAIR values for each single-age group to obtain a column for each of the 55 single ages in the study. For each column, we used the relative fraction of total AAIRs summed over 20 years as an annual factor to multiply each of the 55 year-age groups shown in the first diagnostic column (year 2002).

### 2.3. Interpolation of AAIR Values

AAIR/100,000 is published as mean values assigned to the middle of each 5-year age group (quintet); for example, in quintets 55–59 and 60–64, the mean AAIR values are assigned to years 57 (57.5) and 62 (62.5). These authentic AAIR values (“anchors”) surround 5 blank years, which we filled with interpolated values dictated by the surrounding anchors. The intervals between neighboring anchors were divided into 5 equal AAIR values, sequentially reduced from the larger to the smaller anchor. This generated a pre-factored column for each age group, subsequently multiplied by each of the 20 AAIR percent fractions over 20 diagnostic years to complete a full AAIR matrix. To this end, the matrix represents rows of age cohorts in which every diagnostic year of the same ages, born in different years, shows a continuous annual decline in PD AAIR.

### 2.4. Conversion of Age Cohorts to Birth Cohorts

If a health policy is implemented in a distinct calendar year and affects distinct age groups, local changes in incidence of certain diseases may emerge epidemiologically in the future, reflecting past health policies. For example, BCG vaccination of newborns may theoretically influence the incidence of neurodegenerative and other diseases when relevant groups age. This requires conversion of age cohorts to birth cohorts, in our case, to detect an abrupt change in PD incidence, by identifying the relevant birth cohort/s relating to the beginning or phaseout of the BCG vaccination policy. We pooled down the column of diagnostic year 2020, one year below that of 2021, and sequentially each preceding column, obtaining jagged rows of ages younger by the years from 2021 back to 2002. Now, all ages within each row belong to the same birth cohort (Table S1).

### 2.5. Comparison of the BCG Vaccination Policy with the Non-Vaccinated Controls

**Figure 1:**
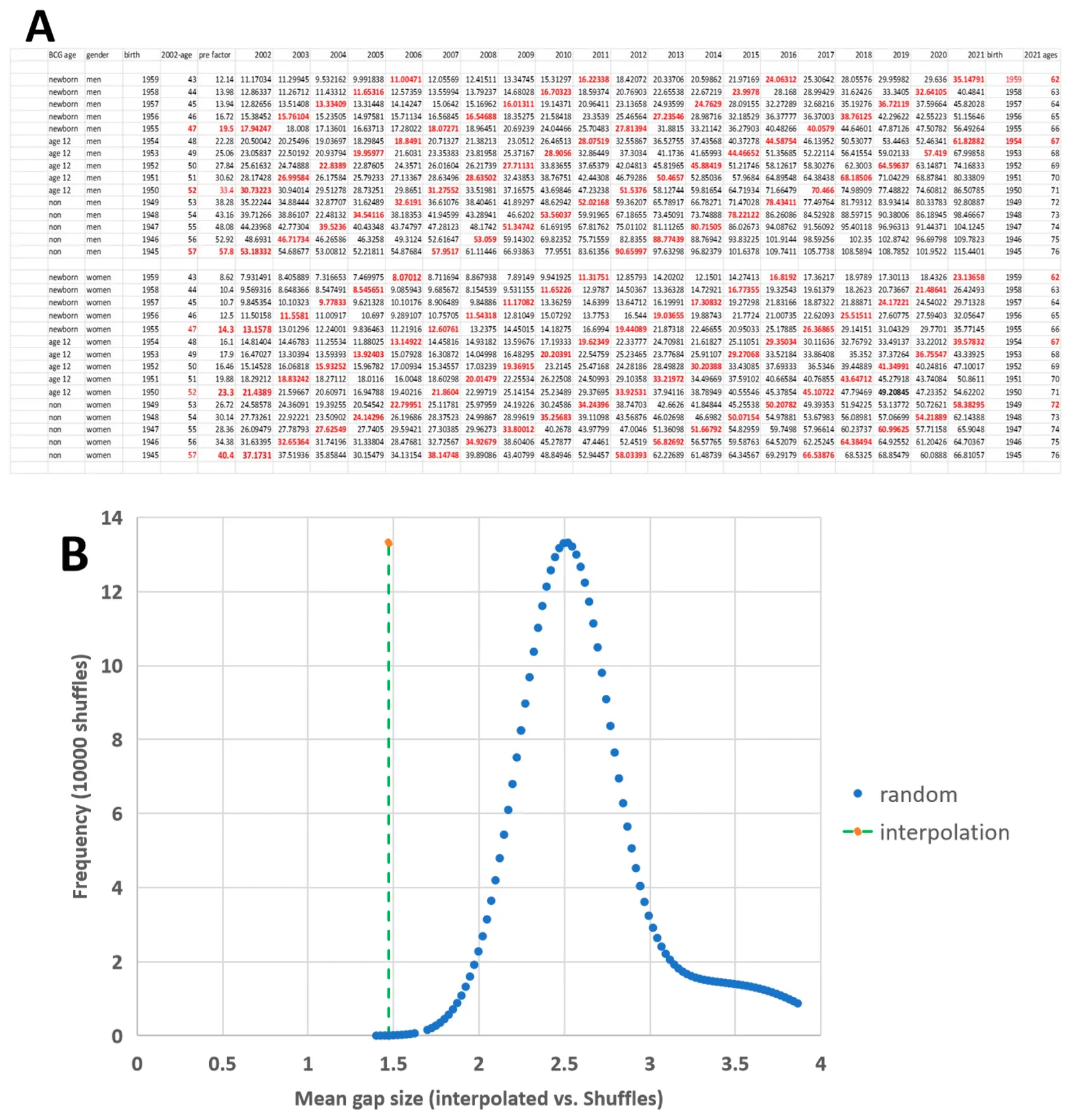
Selected matrix of single-year birth cohorts generated by interpolation of missing values between actual PD AAIR values from Balash et al., showing quintets of 5 age groups, each presenting their mean age-adjusted incidence rates (AAIRs) assigned to the mid-age quintet year. These were arranged in a column across the upper row, indicating 20 single diagnostic years (2002–2021), with total AAIR values that encompassed all age groups (**A**). Submatrices of men and women were selected according to three birth periods, distinguished by different vaccination policies, to investigate the impact of BCG vaccination policies on PD incidence. BCG vaccination of newborns was implemented from 1955 to 1981, of which the 1st 5 years (1955–1959) are included here. From 1962, 7th-grade school pupils were subjected to the Mantoux skin test and were BCG-vaccinated if responses were smaller than 10 mm in diameter. This generated the category of age-12 vaccinated among birth cohorts born from 1950 to 1954. Cohorts born between 1945 and 1949 are the non-vaccinated controls. The published AAIR values in column 4 (red colored “pre-factor”) served as “anchors” between which interpolated values are inserted. The pre-factor mean AAIR values and interpolated values were factored by the percent of total AAIR summed from Balash et al. [25]. This is reflected by the jagged red color values representing factored published AAIRs intermingled with factored interpolated values, which were shuffled randomly 10,000 times between fixed “anchors” and presented in a Gaussian distribution (**B**). The distribution of the interpolated AAIR values is the green perpendicular line far from the Gaussian peak (*p* = 0.01), indicating that it is highly unlikely to be generated by random interpolation.

**Figure 3:**
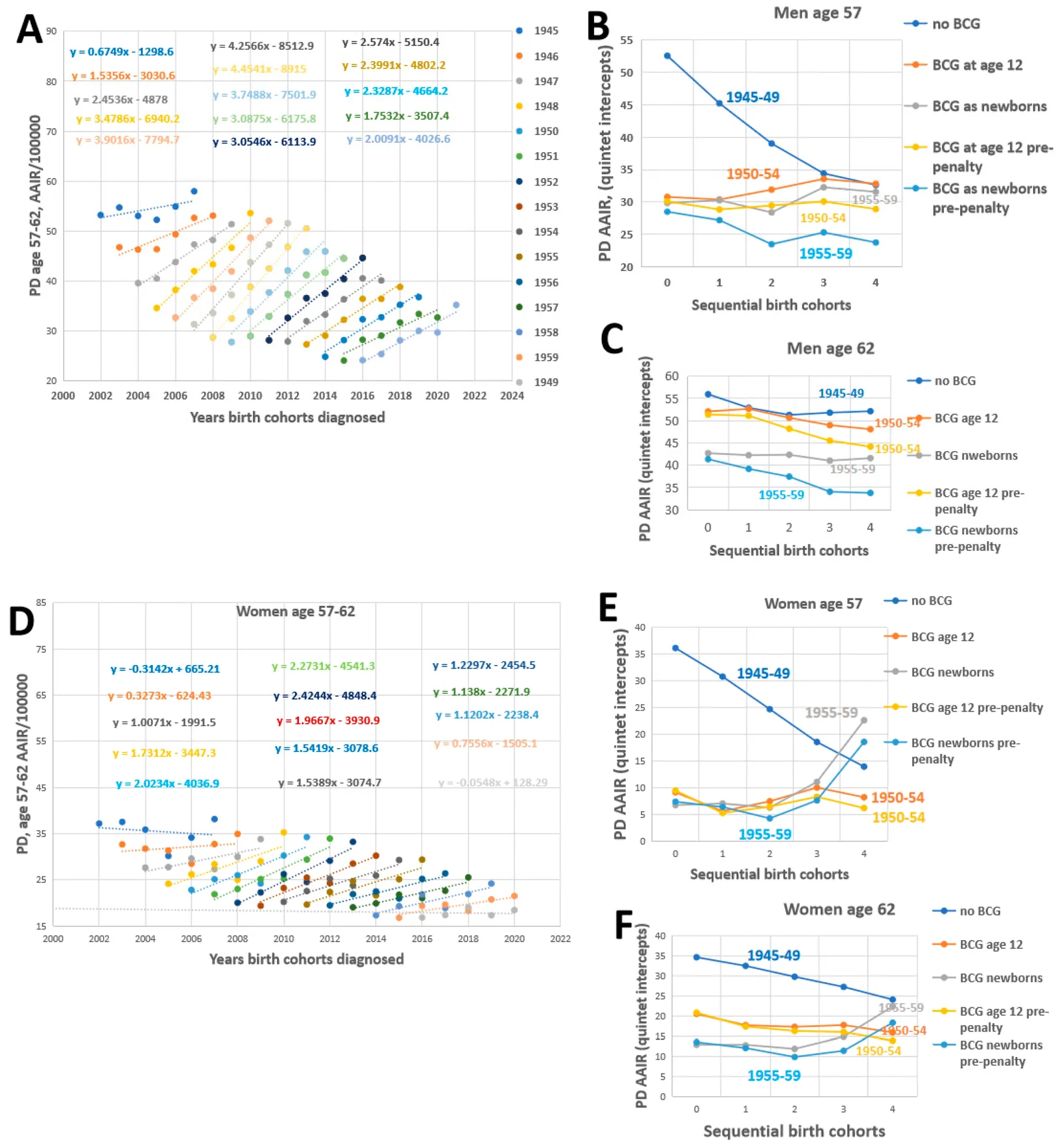
Log regression of PD AAIR values of 3 categories of birth cohorts: The control (pre-BCG vaccination period) birth cohorts born between 1945 (point X = 0 on the x-axis) and 1949 (point X = 4, year-5) were spread over the regression lines. Log regression lines consisted of 5-year intervals between authentic AAIR values (anchors) surrounding interpolated values and were computed over the indicated diagnostic years (x-axis). A total of 15 regression lines, 5 of each vaccination policy for men (**A**) and women (**D**). Least-squares fitting of Figure 1A AAIR data provides the “m” values of the slopes (y = m(x) + b); each regression line runs between the ages of 57 and 62. The slopes were transformed to fit the calendar years (t) corresponding to the diagnostic years of ages 57 and 62 for men (**B**,**C**) and for women (**E**,**F**), (y = [t × m] + b). The slopes of each of the 5 unvaccinated control regressions were added to the corresponding experimental AAIR points as a penalty, compensating for the environmental drift advantage gained by the later-born cohorts (5 years for age-12-vaccinated and 10 years for those vaccinated as newborns). Note the PD differential incidence in men vaccinated at age 12, aged 57 (**B**), versus 62 (**C**), relative to the controls, as opposed to that in women aged 57 (**E**) versus 62 (**F**).

PD is a disease of aging and requires comparing identical age groups from past BCG vaccination policies to remove the time effect of biological amortization (body wear and tear) occurring over a lifetime. Biological amortization is a negative factor underlying the increase in PD incidence. A counteracting factor that favors the reduction in PD incidence, reflecting the decline in PD incidence in Israel, is perhaps related to the general improvement of living conditions (“modernization”) expressed by linearly declining annual PD AAIR values. The birth cohorts of age 12 and the newborn vaccination policy period give an advantage of living 5 and 10 years, respectively, over the control pre-vaccination policy. To compensate for the control of the unvaccinated aged groups born 1945–1949, we calculated their environmental drift using linear regression of the incidence values (PD AAIR) of 5 control birth cohorts diagnosed between 2002–2007 and 2006–2011 (Figure 1A). Each regression line was arranged between the same sequential intervals, 0, 1, 2, 3, and 4, expressing “X” values, from y = m(X) + b; the intercept on the y axis (X = 0) represents, for example, the AAIR controls at age 57 (born in 1945) for each of the 5 sequential calendar years (X = 4 born in 1949). Regression lines between the ages of 57 and 62 are overarching interpolated values that are excluded from comparative calculations (Figure 3A,C). The “m” (in 15 formulas) represents the slope, and “b” the intercept; their values are color-coded and presented as 3 groups, one for each vaccination policy category (upper panel of Figure 3A,D). AAIR values of ages 57 and 62 flanking regression lines, controls, and the vaccination policy categories were separated to form cross-regression lines, as mentioned above. These linear regressions are obtained between AAIR authentic values from ages 57 and 62. The intercepts obtained against the 0–4 positions on the *x*-axis had to be transformed to AAIR values that fit calendar years (“t”) at which actual diagnosis of PD was made. Thus, y = (t × m) + b, where t stands for calendar years of ages 57 and 62 at the extremities of the regression lines (lower panels, Figure 3A men, and Figure 3D women). To this end, interpolated AAIR values are excluded, and values representing ages 57 and 62 are derived from the authentic published mean AAIR arranged as regression lines (Figure 3B,C,E,F). The mean environmental drift is determined annually by the growth of the “X” value, which grows annually by 2.5% of the incidence of the unvaccinated controls (Table S2). It is added to the incidence of vaccinated groups aged 57 and those aged 62 as a penalty for living in 5-year- and 10-year-more-modern living conditions than their respective controls.

### 2.6. Statistical Analysis

**Figure 4:**
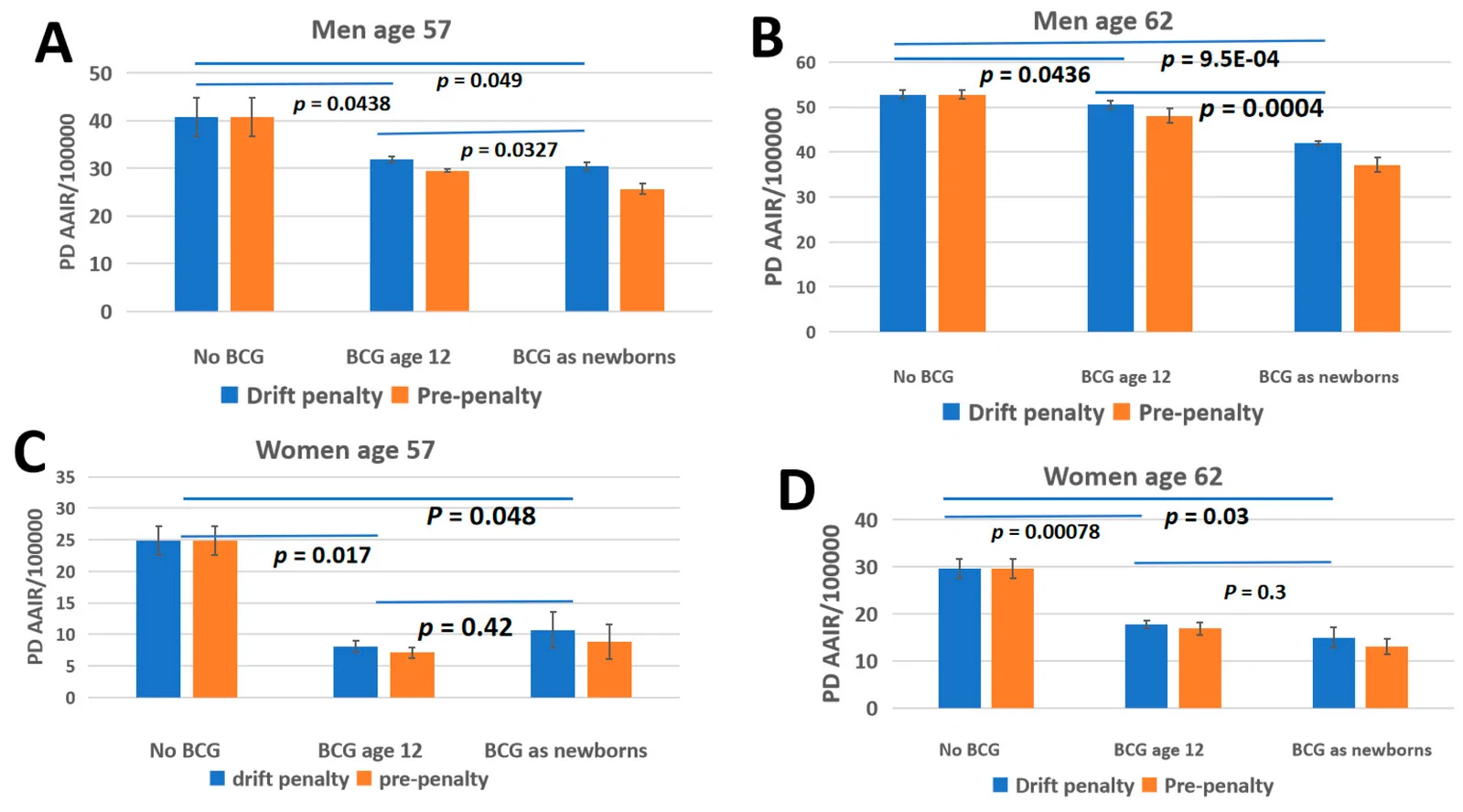
Mean PD incidence of combined birth cohort categories: Mean PD incidence value comparison between identical age 57 men (**A**) and women (**C**) versus age 63 men (**B**) and women (**D**); the values between environmental drift (blue) are derived from unvaccinated controls and added to vaccinated groups as a penalty. Note the advantage of newborn versus age 12 vaccination in lowering PD incidence, compared to women, where both vaccination categories have both a similar and stronger reduction in PD incidence.

The difference between controls and groups born under the two BCG vaccination policy periods was computed using a two-tailed *t*-test (Figure 4). Shuffling of interpolated data between the “anchor” values was done by AI-provided Python code. Ten thousand random rounds of (Monte Carlo) shuffling generated a Gaussian curve (see Figure 1B).

## 3. Results

### 3.1. Deployment of Anchors and Interpolated Trajectories of Birth Cohorts

**Figure 2:**
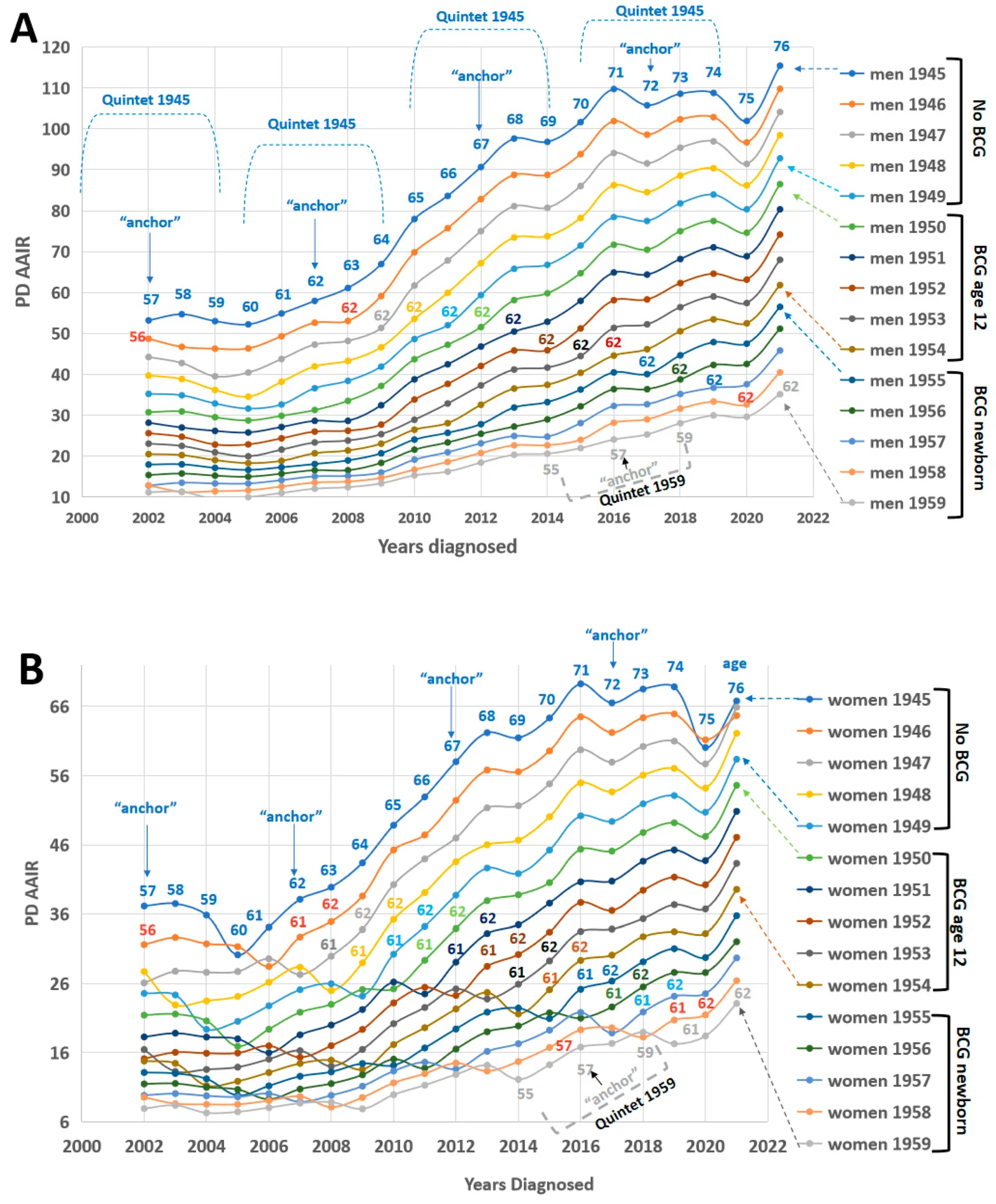
Deployment of PD birth cohort trajectories of three age categories: AAIR values of single-age categories (Figure 1A) depict the mean AAIR values diagnosed annually over 20 years (2002–2021). The cohorts were category a, born between 1945 and 1949, which is before the newborn BCG vaccination policy; category b, born between 1950 and 1954 and vaccinated at age 12 at school; and category c, born between 1955 and 1959, and BCG-vaccinated as newborns. Birth cohorts of men (**A**) and women (**B**) are color-coded, and age groups are indicated above the 1945 trajectory. Age group quintets are indicated by overarching stippled bridges (**A**); the mid-age (anchors) denote the published mean AAIR values of each quintet; and the intervening values are interpolated and subjected to a sensitivity test (Figure 1B). The real AAIR values of mid-ages 57 and 62 of the 55–59 and 60–64 quintets were isolated from all trajectories for analysis of how they reflect the past vaccination policies of categories a, b, and c. Note that trajectories entering age 60 show an upsurge of the PD AAIR, whose slopes are attenuated, reaching vaccination policy periods (categories b and c).

The matrix of AAIR (published and interpolated) values (Figure 1A) is presented as a map of color-coded birth cohort trajectories, with the three categories of past BCG vaccination policies depicted on the right. The corresponding AAIR values plotted against the 20 diagnostic years in men (Figure 2A) and women (Figure 2B) suggest that, from age 60, the PD incidence slope increments are progressively attenuated over the diagnostic years. This is more prominent in women’s trajectories, with upsurge points at ages 50 and 55 that correspond to the age group outlining the quintet borders and are derived from the interpolated AAIRs dictated by the published anchor values. Ages 57 to 62 anchors are the only ones fully included in the Figure 1A matrix, representing age cohorts crossing the birth cohort trajectories. Given that the AAIR of anchor values is authentic, we used them to delineate the slopes between ages 57 and 62 over the 20 diagnostic years. Note that the interpolated values are used to provide the shape of the birth cohort trajectories (Figure 2) and not for incidence calculations.

### 3.2. Comparison of PD Incidence (AAIR) Under 3 BCG Vaccination Policies

Figure 3 shows the differences between five identical age-years for each BCG vaccination policy, grouped into men (Figure 3A–C) and women (Figure 3D–F). Fifteen regression lines, five for each of the three BCG vaccination policies, with indicated slopes and intercepts for men (Figure 3A) and women (Figure 3D), delineated by ages 57 and 62, surround interpolated AAIR values of intervening ages. Each age quintet has five AAIR values of ages 57 and 62; those of the unvaccinated controls (born 1945–1949) show an annual decline in AAIR values. The decline represents the environmental drift used as a penalty added to the corresponding experimental age groups born 5 years later, and a double of the same drift is added to those born 10 years later. PD incidence in men aged 57 and born in 1950, 51, and 52 (age-12-vaccinated) is far below that of the controls born in 1945, 46, and 47, and PD incidence in men born in 1955–1959 is further but only slightly decreased (Figure 3B). In contrast, for the PD incidence in men aged 62, the drift penalized those born during the age 12 vaccination policy period, which is closer to that of the controls; moreover, the drift penalized men born during the years of 1955–1959 (newborn vaccination policy, Figure 3C), with values substantially lower than the values from the age 12 vaccination policy period. This suggests that whatever increases the PD incidence in men aged 62 encounters resistance in men born during the newborn BCG vaccination policy. PD incidence in women aged 57 is far below that of the controls during both vaccination policy periods (age 12 and newborn BCG vaccination). The PD incidence in women aged 62, born under the age-12 BCG vaccination policy, is higher than in those born under the newborn BCG vaccination policy, but far below the incidence in the controls. This suggests that puberty physiology at age 12 confers superior resistance to PD AAIR upsurge at age 62 as opposed to a lack of resistance in pre-pubertal men of the same birth category (age 12 BCG vaccination policy). A prominent feature of the control slopes in both genders is the gradual descent of AAIR values over the progression of the policy (“non-policy” control) period at age 57; at age 62, the AAIR slopes are flattened as the AAIR of the years 1946–1949 gain higher AAIR values approaching the 1945 AAIR (intercept) level. It is as if the environmental drift from each of the control years is penalizing itself at age 62. Furthermore, female pubertal physiology is necessary but insufficient to counteract the age-62 upsurge in PD incidence; the addition of birth during one of the two periods of BCG vaccination policies accounts for its association with resistance to the age-62 upsurge in PD incidence. This is consistent with females’ sex gestalt having a higher (than male) impact on innate immunity independent of sex hormones [26].

### 3.3. Summation of the Vaccination Policies According to Sex Effects

Figure 4 presents the mean AAIR values of BCG vaccination policy periods at ages 57 and 62 for men (Figure 4A,B) and women (Figure 4C,D). The PD mean incidence of age-12 vaccinated men is close (95%) to the mean incidence of controls (Figure 4B, age 62, *p* = 0.0436), compared with women; PD incidence is 59.5%, which is far below the control level (Figure 4D, *p* = 0.00078). At age 57, the mean PD incidence in men vaccinated at age 12 is 80% of the control incidence (Figure 4A, *p* = 0.0438), and in women it is 32% of the control incidence (Figure 4C, *p* = 0.017). In men aged 57 born during the newborn BCG vaccination policy period, PD incidence is 75% of the control incidence (*p* = 0.049); in women aged 57 in this category, PD incidence is only 40% of the control mean incidence (*p* = 0.048). PD incidence in men aged 62 and born during the newborn vaccination period is 79.5% of the controls, as opposed to women in this category, where PD incidence is only 50% of the control incidence. In conclusion, men born during the newborn BCG vaccination policy show a reduction in PD incidence at age 57; in women, the reduction in PD incidence in this category is almost 2-fold compared to men. Resistance to the PD incidence upsurge at age 62 is favored by a combination of female physiology with birth during a mandatory BCG vaccination policy at an early age.

## 4. Discussion

The gap between control slopes and those of vaccination policy periods after penalty by control environmental drift is the basis for the hypothesis that early-age BCG vaccination is robust enough to reduce the development of PD. The method by which this assertion is claimed requires discussion of the limitations. The slopes are based on the authentic values (not interpolated values).

### 4.1. Limitations

When molecular biologists are willing to go out of their way and assess the feasibility of their hypothesis via disease records, they are bound to encounter the hurdle of accessing an inaccessible (existing) database. This required the use of population studies of PD incidence conducted by epidemiologists, which usually combine 5–10 age groups and several diagnostic years. In one source, the diagnostic years were provided as separate incidence values for each year and not combined (i.e., Balash et al.) [25]. The age-adjusted incidence rates posed another limitation, using 100,000 individuals as a denominator, which does not distinguish locally born individuals from immigrants in an unknown PD population whose birth country is not accounted for. In addition, 1/10 of local births are not accounted for by the fact that BCG vaccination was not implemented in the Jerusalem district; it is thus possible that if the present hypothesis is valid, more Jerusalem-born individuals may be included among PD patients relative to the rest of the county’s districts, where BCG vaccination was practiced. Actually, this point is probably a strength rather than a limitation; had Jerusalem births been included in the vaccination policy periods, the PD AAIR might have been even lower for those born under BCG policy periods. Although the following consideration is not a limitation, it should be noted that the start of the Mantoux skin testing for reaction to the BCG vaccine (or exposure to tuberculosis) in the 7th grade (age 12 and in some 13) at school was implemented in 1962, which is seven years after the start of newborn vaccination (in 1955 [27]). This implies that during the 5 years preceding 1955, there were birth cohorts vaccinated at age 12, covering earlier births and some children of immigrants. However, the birth cohorts 1950–1954 are special as they may support the age-12 difference between girls vaccinated during the pubertal process compared to boys at age-12, who are still pre-pubertal. However, despite the pubertal age difference, the strong reduction in PD incidence in women born during the age-12 vaccination policy (versus a smaller reduction in age-matched men) was not significantly different from the reduction in those vaccinated as newborns. This implies that female sex gestalt is a more important factor than sex hormones during puberty for reduction in PD incidence by early age BCG vaccination. This is consistent with females’ sex gestalt having a higher impact than males on trained innate immunity independent of sex hormones [26].

### 4.2. Limitations of the Supporting Study of PD Decline in Germany by Fink et al. [23]

The limitations claimed by the German study (although interpolation results are not presented here) stem from the unknown ratio of AOK health system registrants born in West versus East Germany. The East German BCG vaccination program was extended in 1951 and 1952; the % of coverage is not disclosed. However, starting from 1953, BCG vaccination in the East became mandatory for all newborns. Thus, the 1953 birth cohort and later, guarantees that whoever was born in East Germany during these years and registered with the AOK health system (after Germany unification) provides a population background of relative PD resistance (if the present hypothesis is valid), while the rest of the registrants (born in West Germany) contribute only 20–25% coverage of BCG vaccination at birth [24].

The limitations are due to the reconstruction (interpolation) of missing incidence values for the single-age cohorts derived from the mean incidence published by the original authors for 5-year groups (“quintets”). The interpolation for the study of Balash et al. [25] required an upward or downward trend within each age-year quintet, dictated by the mean incidence of the neighboring quintet. If the natural data of the hidden incidence trend line for separate single-age groups present peaks and valleys, by combining these groups into quintets, then their summed-up incidence acts as a mean trend line for which the present distribution acts as a moving incidence average that cuts through the putative unreported peaks and covers the valleys, which is not less legitimate than the unified incidence of the original author’s quintets. Yet a sensitivity test was performed by shuffling 10,000 random interpolations parallel with the calculated interpolations, demonstrating that they are unlikely to result from random shuffling (Figure 1B, *p* = 0.01).

### 4.3. How Could the BCG Vaccine Protect Against PD?

If the protection of brain tissue from as early as newborn vaccination by a direct central effect of the vaccine is to take place, one would expect PD pathology to be already active immediately postpartum. Interestingly, BCG vaccination in newborns has been shown to protect at age 62, not in non-vaccinated controls, which is a phenomenon possibly related to non-linear aging indicated by a sudden reduction in the expression of a group of genes around age 62 [28]. This phenomenon should be evaluated for a possible connection to PD. Alternatively, activation of the immune system by BCG vaccination is already known to occur by at least two mechanisms. One is the training of innate immune cells [29] of the myeloid arm, involving emergency granulopoiesis [27,28] and the secretion of cytokines that, in turn, stimulate bone marrow progenitors and hematopoietic stem cells, and epigenetically imprint them to produce trained progeny [29,30,31]. It is believed that training in vaccinated newborns is more efficient than in adults [32,33,34,35], supporting the importance of the present analysis. In mice, intravenous (I.V.) vaccination is more efficient than by the intradermal route [29]. Neutrophil granulocytes are efficient at killing tumor cells via reactive oxygen species (ROS) as tumor-associated neutrophils (TANs) [36]; however, it is unimaginable that ROS-secreting TANs protect the brain against neurodegeneration. Interestingly, TAN3 (N2 neutrophils) are tumor-induced neutrophils that accumulate around tumor cells, protecting the tumor by attracting angiogenesis and promoting tumor survival [37,38]. Experiments in the NMIBC model in mice demonstrated that β-glucan added to BCG therapy increased TAN1 and TAN2, and decreased TAN3, resulting in 100% tumor eradication [39]. It is thus intriguing to suggest that NMIBC adult survivors of bladder tumors treated with BCG alone may have had a free population of TAN3 cells that migrated to the brain, and thus enhanced angiogenesis in the brain, which reduced the incidence of PD and AD. In addition to neutrophils, the proliferation of monocytes and monocyte-derived macrophages of the myeloid innate immune arm is stimulated by BCG [30,40]. These cells have been implicated in protecting against neurodegeneration [13,41,42,43]. In the mouse model, where BCG was injected via I.V., innate immune cells (myeloid arm) were more abundant than adaptive immunocytes of the lymphoid arm [29,44]. However, BCG antigens can activate adaptive lymphocytes, such as T cells, whose activation is associated with the conversion of anaerobic to aerobic glycolysis [45], as well as innate immune cells [46,47,48]. Thus, BCG vaccination generates a metabolic effect in immune cells reminiscent of the Warburg effect, similar to that of tumor cells, which, in the presence of tumor cells and immunocytes, poses them as competitors for glycolytic-derived energy.

### 4.4. Metabolic Connection of BCG to Neuroprotection

BCG vaccination has induced aerobic glycolysis in immune cells, reducing insulin requirements in insulin-dependent diabetic patients [49]; however, this requires an induction period of 3 years, which is a phenomenon also demonstrated by the gradual increase in specific protein expression in T regulatory cells [46]. The attenuation of hyperglycemia was explained by a substantial upregulation of glycolytic rate and epigenetic reprogramming, resulting in increased glucose influx into immunocytes. Thus, in the absence of tumors, BCG vaccination may (hypothetically) endow immune cells with the ability to protect neurons from the loss of efficient energy sustainment by microglia and astroglia. Neurons, as opposed to astrocytes, are inefficient in carrying out glycolysis due to poor expression of an enzyme that maintains the dual phosphorylation of fructose [50]. Instead, neurons express monocarboxylate transporters for lactic acid/pyruvate [51,52], relying on astrocytes for their abundant supply and converting lactate to pyruvate and acetoacetate, joining the neuronal Krebs cycle. This way, neurons depend less on their own glycolysis and gain their end product for oxidative phosphorylation. Therefore, astrocytes are essential for supplying neurons with energy [51], whose senescence can contribute to neurodegeneration. To this end, increasing the glycolytic rate in peripheral immunocytes by BCG vaccination may sustain [52] and replace astrocytes at least for running the glycolytic pathway, providing its end product to neurons. Whether this connection to BCG is feasible is yet to be demonstrated experimentally.

### 4.5. A Possible Involvement of BCG in Immune Checkpoint Expression Contributing to Neuronal Metabolism

Roughly one-half of NMIBC patients treated with BCG relapse due to the expression of the PD-1 receptor on PBMCs and its PD-L1 ligand on tumor cells [53,54], conferring a metabolic advantage to the tumor in competing for energy against the immunocytes [55]. Checkpoint blockade by specific antibodies may lead to internalization of PD-L1 by tumor cells and abrogate the high glycolytic rate induced by the PD-L1-associated signalosome [52]. Initially, in PBMCs, BCG increases PD-L1 expression more than PD-1 [53], which is recruited by the short intracellular PD-L1 motifs [56]. Later, in some patients, PD-1 expression may increase, while in others, PD-L1 ligand expression may be downregulated in PBMCs (not in the tumor cells) [57]. Treatment of an AD mouse model with checkpoint blockade improved cognition [42], while in humans, it caused neurological side effects [58]. Hypothetically, the protection of tumor resistance via PBMCs expressing PD-1 bound to tumor-expressed PD-L1 may exemplify the protection of brain tissue by its expression of PD-L1 bound to PBMC-expressed PD-1 via BCG therapy [57]. BCG treatment was shown to improve cognitive function in humans [20]. Hypothetically, brain-infiltrated PBMCs may protect brain cells via checkpoint bonds. This hypothesis can be stated as a rule of thumb: “protecting tumor cells by PD-1 expressing immunocytes in the case of immune failure to kill protects the brain against neurodegeneration by similar immunocytes.”

## Data Availability

The original contributions presented in this study are included in the article/Supplementary Material. Further inquiries can be directed to the corresponding author.

## Supplementary Materials

The following supporting information can be downloaded at https://www.mdpi.com/article/10.3390/biomedicines14091945/s1. Table S1: Men and women birth cohorts; Table S2: calculation of environmental drifts.

## Abbreviations

BCG	Bacillus Calmette–Guerin
PD	Parkinson’s disease
AAIR	Age-adjusted incidence rates
NMIBC	Non-muscle invasive bladder cancer
AD	Alzheimer’s disease
UPR	Unfolded protein response
ER	Endoplasmic reticulum
ROS	Reactive oxygen species
TANs	Tumor-associated neutrophils
I.V.	Intravenous
PD-1	Protein death-1
PD-L1	PD-1 ligand-1
PBMCs	Peripheral mononuclear cells
